# Minorities’ Diminished Returns of Parental Educational Attainment on Adolescents’ Social, Emotional, and Behavioral Problems

**DOI:** 10.3390/children7050049

**Published:** 2020-05-18

**Authors:** Shervin Assari, Shanika Boyce, Cleopatra H. Caldwell, Mohsen Bazargan

**Affiliations:** 1 Department of Family Medicine, Charles R Drew University of Medicine and Science, Los Angeles, CA 90059, USA; Mohsenbazargan@cdrewu.edu; 2 Department of Pediatrics, Charles R Drew University of Medicine and Science, Los Angeles, CA 90059, USA; ShanikaBoyce@cdrewu.edu; 3 Department of Health Behavior and Health Education, School of Public Health, University of Michigan, Ann Arbor, MI 48109-2029, USA; cleoc@umich.edu; 4 Center for Research on Ethnicity, Culture, and Health, School of Public Health, University of Michigan, Ann Arbor, MI 48109-2029, USA; 5 Department of Family Medicine, University of California, Los Angeles (UCLA), Los Angeles, CA 90095, USA

**Keywords:** ethnic groups, socioeconomic status, socioeconomic factor, parental educational attainment

## Abstract

**Aim:** To compare racial groups for the effect of parental educational attainment on adolescents’ social, emotional, and behavioral problems. **Methods:** In this cross-sectional study, 10,762 youth from the Adolescent Brain Cognitive Development (ABCD) study were included. The independent variable was parental educational attainment. The main outcomes were (1) anxious and depressed mood, (2) withdrawn and depressed affect, (3) somatic complaints, (4) social and interpersonal problems, (5) thought problems, (6) rule-breaking behaviors, (7) attention problems, and (8) violent and aggressive behaviors. These scores were generated based on parent-reported behavioral problems measured using the Child Behavior Checklist (CBCL). Race and ethnicity were the moderators. Linear regression was used to analyze the ABCD data. **Results:** Overall, high parental educational attainment was associated with lower scores across all domains. Race and ethnicity showed statistically significant interactions with parental educational attainment on adolescents’ fewer social, emotional, and behavioral problems (all domains), net of all confounders, indicating smaller tangible gains from their parental educational attainment for Black and Hispanic compared to non-Hispanic White adolescents. **Conclusions:** The protective effects of parental education against social, emotional, and behavioral problems are systematically diminished for Hispanic and Black than non-Hispanic White adolescents.

## 1. Introduction

The Child Behavior Checklist (CBCL), also known as the Achenbach System of Empirically Based Assessment, is one of the most widely used tools for screening social, emotional, and behavioral problems in adolescents [1]. The CBCL instrument uses a parental report form to screen for social, behavioral, and emotional problems. The CBCL is commonly used across settings including but not limited to schools, medical settings, mental health facilities, child and family services, health management organizations, and public health agencies [2]. It has been used by thousands of published scholarly articles [2]. CBCL has shown high content, structural, concurrent, and criterion validity for the measurement of behavioral outcomes in racial and ethnic minority youth. CBCL is also shown to have high cross-cultural validity, which makes it a useful tool to compare outcomes across racial and ethnic groups [3]. 

The CBCL generates scores on the following eight domains: anxious and depressed mood, withdrawn and depressed affect, social and interpersonal problems, somatic complaints, thought problems, attention problems, violent and aggressive behaviors, and rule-breaking behaviors. The CBCL results closely correlate with the Diagnostic and Statistical Manual of Mental Disorders based diagnoses [4] such as anxiety disorder, oppositional defiant disorder, conduct disorder, somatic disorder, affective and mood disorders, as well as attention deficit disorder. Many studies have established high validity as well as reliability of CBCL too [5].

Relative to non-Hispanic Whites, Hispanic, and Black youth are at an increased risk of social, emotional, and behavioral problems [6]. Black and Hispanic youth report more externalizing and internalizing symptoms than their White counterparts [6]. As a result of this gap, we observe racial and ethnic inequalities in substance use [7], aggressive behaviors [6], conduct disorders [6], anxiety [6], depression [6], and academic achievement [8]. Some of these problems can operate as a gateway to future economic and health problems later in life [9,10,11,12]. Thus, these early inequalities cause future inequalities later in life [9,10,11,12].

Among various social factors, in addition to race/ethnicity, socioeconomic status (SES) such as parental education [13] are among major social determinants of early youth outcomes. Highly educated parents report higher levels of parental involvement, which has consequences across domains of youth development [14]. Some recent evidence, however, suggests that relative to non-Hispanic Whites, Black and Hispanic youth show weaker effects of parental education on tangible youth outcomes, also known as Minorities’ Diminished Returns (MDRs) [15,16]. While for the first time, MDRs were reported by Ferraro, it was further theorized and explained by an extensive work by Assari. Although MDRs are predominantly shown for Blacks, they are replicated for almost every marginalized group such as Hispanics, Asian Americans, Native Americans, Immigrants, and even marginalized White people. These MDRs also seem to be robust as they hold regardless of SES indicator and outcome, setting, and age group. 

While parental education (i.e., highest education level of parents) is a predictor of a wide range of positive developmental and health outcomes of youth across domains [17,18,19,20,21,22,23,24]. Youth with highly educated parents are less likely to experience economic adversities, stress, behavioral problems, and poor health [21,22,23,24]. These families are more likely to have the resources that prevent adversities. They also have access to the resources that help them manage these adversities when they are faced. As a result, at least some of the racial and ethnic gaps in adolescent outcomes are attributed to the lower parental educational attainment of racial and ethnic minority families such as Blacks and Hispanics [22,25,26]. As such, enhancing education levels of racial and ethnic minorities is being regarded as the main strategy to close the racial and ethnic inequalities that children and adolescents are experiencing [13,20,27,28].

As shown by the MDR literature, educational attainment of own [29] and parents [20,21,28] may generate unequal outcomes for the members of diverse racial and ethnic groups. Racial and ethnic minority groups may differently be able to navigate resource systems or their resources to secure tangible outcomes in the presence of high educational attainment [16,28,30,31,32,33]. For example, Black and Hispanic youth show weaker effects of parental educational attainment on various outcomes relative to their non-Hispanic counterparts [15,16,31,34,35]. 

Educational attainment of self and parents, however, differentially translates to tangible outcomes for Hispanic, Black, and non-Hispanic White families [15,16]. Among adults, own educational attainment has smaller protective effects on the risks of smoking [36], drinking [37], poor diet [38], obesity [35], depression [39], suicidality [40], and mortality [41] for Black and Hispanic than non-Hispanic White people. There are even some evidence linking high SES to poor mental health for Black adolescents [42] and adults [39], in terms of depression [39], and suicidal ideation [40]. Some of the undesired mental health outcomes of Black and Hispanic families is because high SES Black families are within greater proximity to non-Hispanic White families, which increases their discriminatory experiences [43,44]. Discrimination is linked to multiple developmental and health outcomes [45,46,47], including but not limited to poor educational performance [13]. However, there is a dearth of evidence about race and ethnicity moderating parental effects.

The literature on MDRs has shown that diminished returns of SES indicators such as parent education are among the overlooked mechanisms by which transgenerational racial and ethnic disparities emerge [31,34,35]. In several studies [31,34,35], family SES shows stronger effect on adolescents body mass index (BMI) [35], self-rated health (SRH) [34], attention deficit hyperactivity disorder (ADHD) [48], mental health [28], impulse control [31], and school attachment [49], for White than Black families. In some recent studies, parental educational attainment also more strongly improves educational attainment [20], school performance [13], and school bonding [49] of non-Hispanic White than Black and Hispanic adolescents. 

### Aims

To extend the existing knowledge on the MDRs literature, we compared racial and ethnic groups for the effects of parental educational attainment on adolescents’ social, emotional, and behavioral problems. We expected weaker effects of parental educational attainment on youth social, emotional, and behavioral problems for Black and Hispanic than non-Hispanic White families. 

## 2. Methods

### 2.1. Design and Settings

This was a secondary analysis of the Adolescent Brain Cognitive Development (ABCD) study [50,51,52,53,54]. This was a cross-sectional analysis of the ABCD data. ABCD is a national, state-of-the-art brain imaging study of youth brain development. More information about ABCD’s purpose, methodology, and measurement is available elsewhere [50,55].

### 2.2. Advantage of the ABCD Study

The advantages of using the ABCD dataset were (a) national sample, (b) large sample size, (c) large sample of African Americans and Hispanics, (d) publicly available data, and (e) considerable socioeconomic and behavioral variables [50,51,52,53,54]. 

### 2.3. Participants and Sampling

Participants of the ABCD study were selected across multiple cities across states. This sample was mostly recruited through school systems. A detailed description of the sampling of the ABCD is available here [56]. With one important departure, the ABCD cohort recruitment emulates a multi-stage probability sample of eligible children: a nationally distributed set of 21 primary stage study sites, a probability sampling of schools within the defined catchment areas for each site, and recruitment of eligible children in each sample school. The major departure from traditional probability sampling of US children originates in how participating neuroimaging sites were chosen for the study. Although the 21 ABCD study sites are well-distributed nationally, the selection of collaborating sites is not a true probability sample of primary sampling units (PSUs) but was constrained by the grant review selection process and the requirement that selected locations to have both the research expertise and the neuroimaging equipment needed for the study protocol. As a consequence, neuroimaging research centers are more likely to be located in urban areas, resulting in a potential under-representation of rural youth. The recruitment catchment areas of the 21 participating sites encompass over 20% of the entire US population of 9–10 years old individuals. Moreover, a carefully designed sampling and recruitment process within sites, described below, aims to ensure both local randomization and representativeness while also yielding a final combined ABCD sample that we hope will provide a close approximation to national sociodemographic distribution. The sociodemographic factors on which the sample is recruited include age, gender, race and ethnicity, socioeconomic status, and urbanicity [56].

#### 2.3.1. Study Variables 

The study variables included demographic factors, SES indicators, as well as youth outcomes (social, emotional, and behavioral problems).

#### 2.3.2. Outcome

Using the Child Behavior Checklist (CBCL), also known as the Achenbach System of Empirically Based Assessment, the study had the following eight outcomes: (1) anxious and depressed mood, (2) withdrawn and depressed affect, (3) social and interpersonal problems, (4) somatic complaints, (5) thought problems, (6) attention problems, (7) violent and aggressive behaviors, and (8) rule-breaking behaviors [4]. These CBCL sub-scores closely correlate with the Diagnostic and Statistical Manual of Mental Disorders (DSM-IV-TR) based diagnoses [1]. The CBCL instrument uses a parental report form to screen for social, behavioral, and emotional problems. The CBCL is commonly used across settings including but not limited to schools, medical settings, mental health facilities, child and family services, health management organizations, and public health agencies [2]. It has been used by thousands of published scholarly articles [2]. 

#### 2.3.3. Moderator

*Race.* In the ABCD study, parents reported race and ethnicity; both treated as dichotomous variables (Table 1).

#### 2.3.4. Independent Variable

*Parental Educational Attainment.* Participants were asked, “What is the highest grade or level of school you have completed or the highest degree you have received?” Responses ranged between 1 and 21, with a higher score indicating higher educational attainment.

#### 2.3.5. Confounders

*Age, Gender, and Parental Marital Status.* Parents reported the age of the youth. Age was calculated as the difference between the date of birth to the date of the enrolment to the study. Age was a continuous measure in years. Gender was a dichotomous variable. Parental marital status, a dichotomous variable, was self-reported by the interviewed parent (Table 1).

### 2.4. Data Analysis 

We used the SPSS statistical package for our data analysis. Mean (standard deviation (SD)) and frequency (%) were reported for descriptive purposes. To estimate bivariate analyses between the study variables, we used the zero-order Pearson correlation test. To perform our multivariable analyses, we performed two multiple linear regressions for each outcome. All our models were performed in the pooled sample. These models controlled for age, gender, and parental marital status. *Model 1* was performed with race, ethnicity, parental education, and covariates. *Model 2* included the main effects of race, ethnicity, parental education, as well as two interaction terms between race and ethnicity with parental education. In all these models, a CBCL domain was the outcome. Unstandardized regression coefficient (b), p-value, and sample size were reported for each model. 

### 2.5. Ethical Aspect

The ABCD study protocol is approved by the University of California, San Diego (UCSD) Institutional Review Board (IRB). All participants gave assent. Parents signed informed consent. More detailed information on the ABCD study ethics is available elsewhere [55]. As we used fully de-identified data, our study was non-human subject research. Thus, it was exempted from a full review.

## 3. Results

### 3.1. Descriptives

The current analysis was performed on 10,762 8–11 years old adolescents who were either White (*n* = 8257; 76.7%), Black (*n* = 2506; 23.3%), non-Hispanic (*n* = 9006; 83.7%) or Hispanic (*n* = 1757; 16.3%). Table 2 presents descriptive statistics of the pooled sample.

### 3.2. Unadjusted Bivariate Correlations

Table 3 presents the results of the bivariate correlations. In the overall sample, race and ethnicity were inversely correlated with parental educational attainment. Race was significantly and positively correlated with all CBCL domains. However, ethnicity, was not universally correlated with all CBCL domains. High parental education and being from a married family, however, were inversely correlated with all CBCL domains. Male gender was correlated with all CBCL domains. Age, however, was not universally correlated with all CBCL domains. All CBCL domains were, however, positively correlated with each other (Table 3). 

### 3.3. Multivariate Analysis 

Table 4 shows the results of various linear regression models in the overall (pooled) sample. Each of the two of these models were specific for each outcome. *Model 1* (Main Effect Model) showed positive effects of parental educational attainment on all CBCL domains. *Model 2* (Interaction Model) showed two interaction terms between race and ethnicity with parental educational attainment on CBCL domains, suggesting that the protective effects of parental educational attainment against high scores across CBCL domains are weaker for Hispanic and Black adolescents relative to their non-Hispanic White counterparts. The only non-significant interaction was between ethnicity and parental educational attainment on one of the CBCL domains, namely withdrawn and depressed affect. This means Hispanics and non-Hispanics did not seem to vary for the association between parental educational attainment and this single CBCL domain, namely withdrawn and depressed affect (Table 3).

## 4. Discussion

While overall, there was a positive effect of high parental educational attainment on social, emotional, and behavioral problems in US, these protective effects all depended on race and ethnicity. Higher parental educational attainment was reflective of lower adolescents’ social, emotional, and behavioral problems for non-Hispanic White youth, however, the amount of protection against social, emotional, and behavioral problems due to high parental educational attainment is considerably smaller for Black and Hispanic adolescents. 

The first finding was corroborated by previous studies. We already know that adolescents with highly educated parents do better regarding various social, emotional, and behavioral outcomes. They show less aggression, substance use, depression, and anxiety [17,18,19,20,21,22,23,24]. Our first finding supports the previous studies on family SES as a fundamental cause [57,58]. Similarly, youth outcomes show social gradient and follow SES [59,60,61]. Our findings suggest that high parental education is associated with better youth outcomes across domains. We found lower social, emotional, and behavioral problems in youth with high education. As youth early outcomes are gateways to various outcomes later in life during adulthood [62], the effects of parental education should be regarded seriously.

Our second finding was similar to what the literature has already shown regarding the MDRs of SES indicators particularly parent education for Black and Hispanic compared to non-Hispanic White families and individuals [29,30,63,64]. MDRs are well documented within individuals, within families, thus they contribute to trans-generational effects of inequalities. MDRs are repeatedly shown for almost all SES resources, age groups, outcomes, and marginalization types [15,16]. 

A recent study used some national data and found that Black adolescents receive smaller academic benefits from parental social class than their white counterparts [8]. Authors found that youth from highly educated Black and Hispanic families are less likely to be accepted to college despite high occupational prestige of their parents [8]. They, however, found that parents’ gender and parental marital status play a role in the racial differences in the marginal return of parental SES on adolescents’ outcomes. Two-parent White and Black households did not differ in the return of parental education. For adolescents not in two-parent households, however, Blacks and Hispanics benefit less than non-Hispanic Whites from mothers’ but not fathers’ occupational prestige on their college enrollment [8].

We argue that at least some of the existing MDRs may be due to the fact that education generates worse working conditions for Hispanic and Black parents compared to non-Hispanic Whites [30,65,66]. Due to the existing racism that is rampant in the US labor market, educated Black and Hispanic employees work in worse jobs that are lower in pay and more in stress. Thus, similarly educated families make less income if they are Black or Hispanic [21,67]. Given that racism is a part of the US labor market, Black Americans face more difficulty compared to White Americans in securing a high paying job. As a result, at each educational attainment, Black parents make less income than White parents. The US social system has increased the psychosocial cost of upward social mobility for Black and Hispanic families. Being charged with extra costs for upward social mobility, Black and Hispanic families gain less from their educational attainment. Upward social mobility is much more stressful and less facilitated for racial and ethnic groups, with additional efforts being required by Hispanic and Black individuals than non-Hispanic Whites [15,16]. Historically, Blacks and Hispanics have had less political power, thus, they have had a less say in writing laws and policies [68,69,70]. In the absence of a political voice of Black and Hispanic people, US policies are written by the dominant group, non-Hispanic Whites. These policies have shaped in a way that they have historically maximized Whites’ economic gain, leaving Blacks, Hispanics, and other minority populations struggle with various societal barriers on a daily basis [15,16]. 

At each level of educational attainment, Black and Hispanic parents face disproportionately more stress in their daily lives. Such increased stress may reduce how much parents and students can engage, thus, youth can gain from their available parent education. Highly educated Black and Hispanic families may experience more not less discrimination on a daily basis. Highly educated Black and Hispanics may also be more, not less, vulnerable to interpersonal discrimination. This is in part because highly educated Black and Hispanic families are more likely to be surrounded by White families and attend predominantly White schools and work in predominantly White workplaces, all increasing their exposure to discrimination. We also know that a high level of discrimination reduces the expected gains of educational attainment [71,72,73].

We hypothesize that environmental factors affect school options for youth from high SES Black and Hispanic families. As a result, children of highly educated Black and Latino parents still continue to attend high risk highly segregated schools. Such schools have less funding, have lower quality teachers, and more high-risk classmates and peers who are also from lower SES. This pattern is different than high SES White adolescents who receive education at predominantly White schools.

In addition, Blacks and Hispanics may be ostracized/criticized and guilted from within for being like/aspiring up the economic/social ladder via higher educational attainment. Within group discrimination is well-described and high SES Blacks may be criticized as acting White. In addition, tax is a function of SES, thus, low SES areas would have poor schooling. Such schools are tied to school district funding. Thus, area-level SES factors, which highly differs between high SES Blacks and Whites heavily influence the resources available in schools that White, Hispanic, and Black pupils receive education.

As suggested by the MDRs [15,16], equal SES resources result in unequal outcomes, with marginalized and stigmatized social groups experiencing a relative disadvantage in comparison to the socially privileged group. Although this study specifically looked at the effect of parental education level on adolescents’ behavioral problems, these patterns seem to be independent of age, SES resource, and outcome. More than other SES indicators, however, educational attainment has shown differential effects by racial and ethnic groups, with Hispanics and Blacks benefitting less than non-Hispanic Whites. This occurs not only with educational attainment [29] however, but with employment [74], income [31], and marital status [32]. Educational attainment results in more gain for White than Black adolescents [31,34,35], adults [30], and older adults [37]. Additionally, MDRs not only apply to Blacks [35] or Hispanics [29,75,76,77] as they also hold for Asian Americans [78], Native Americans [79], and LGBTQs [63].

The current study did not explore societal and contextual processes that could explain intergenerational MDRs of education for racial and ethnic minorities. MDRs may be due to institutional and structural racism [15,80]. Marginal returns may also be smaller for families with a history of childhood poverty [81]. Racial prejudice and discrimination may also interfere with the benefit that is expected to follow education [82,83,84]. Thus, multilevel economic, psychological, and societal mechanisms may be involved in explaining racial and ethnic gaps in the returns of parental education [15,80]. 

Black and Hispanic individuals are likely to stay in poor neighborhoods despite high SES. As a result, youth from Black and Hispanic families are more likely to remain at risk of environmental exposures. Similarly, youth from highly educated Black and Hispanic families are also likely to remain at high risk of high-risk peers which are involved in behavioral problems [78,85].

The society and social structure may be key conduits through which MDRs are developed and sustained. As a result, additional attention should be paid to various societal processes that may interfere with the returns of educational outcomes. According to the social reproduction theory, intergenerational educational outcomes may vary across groups [27]. Chetty showed that the intersection of race and gender alters the likelihood of upward social mobility in the US [86].

In this study, we conceptualized race and ethnicity as social rather than biological constructs [87]. As a result, our study is distinct from the behavioral genetics studies that assume that there is causal genetic causation between race/ethnicity and phenotypes, traits, and behaviors [88,89,90]. We did not take a biological approach for this manuscript. We see race and ethnicity as proxies of access to the opportunity structure, treatment by the society, living conditions, history, and access to the power structure. For us, race and ethnicity are reflective of life conditions, as well as racism and discrimination, and legacy of slavery and Jim Crow policies, and oppression [87,91,92,93] not biology [87,91,92,93]. Thus, we remain distant from the arguments on the interaction sociological and biological factors (nature versus nurture) or genes versus environment [88,89,90,94].

Some of the existing racial and ethnic inequalities in the returns of educational attainment (i.e., MDRs) is due to poor quality of education in predominantly Black and Hispanic urban areas. Blacks, Hispanics, and Whites attend schools that are qualitatively different in their quality and resources. In addition to the education system is labor market discrimination. On top of these are institutional and structural racism and colorism that result in differential access of racial and ethnic groups to the opportunity structure [30,95]. US society treats racial and ethnic groups differently, thus, compared to non-Hispanic Whites, Blacks and Hispanics get less chance to mobilize their educational attainment and turn them to outcomes [15,16]. 

## 5. Limitations 

No study is without methodological limitations. The main limitations of this study include cross-sectional design. In the absence of longitudinal data with multiple observations of the same variable (i.e., SES and social, emotional, and behavioral problems), we cannot test causal associations between race/ethnicity, parental educational attainment, and youth outcomes. Similar to most of the literature on MDRs, this study focused exclusively on Blacks, Hispanics, and Whites. We still need more studies on other ethnic groups such as Asian Americans, and Native Americans as well as other marginalizing identities beyond race and ethnicity. Not only race and ethnicity but all marginalizing identities potentially reduce the gains that follow educational attainment [63,76,77,96]. Similarly, this study only investigated MDRs of education. Other research may study other SES indicators such as wealth, income, and parental employment as well as non-economic resources such as coping [97,98]. We intentionally did not control for income because previous research has suggested that income may be why educational attainment generates fewer tangible outcomes for Blacks and Hispanics than non-Hispanic Whites [21,30,67]. Epidemiologists have warned about controlling for a mediator as a source of bias [99,100]. We still do not know if parental higher-level SES indicators such as neighborhood SES and income or racial segregation similarly influence outcomes across race/ethnic groups, or such contextual factors can explain MDRs. Future research may study structural and contextual factors that mediate (explain) and moderate (undo) MDRs of educational attainment. In addition, Blacks and Hispanics are ethnically diverse who differ in nativity, country of origin, and SES. There is a need to study the variation within these ethnic groups. Future research may also expand the work to immigrant and LGBT families. Since the sample size was very large, there is a need to replicate these findings in smaller studies. Such research would confirm if we can generalize these findings. Finally, the beta weights of the significant interactions were not very large. Even if the betas are small, they are observed across domains. That means, regardless of their effect sizes, they have large consequences. Despite the above listed limitations, these results extend the existing literature on the role of MDRs of parental education as an overlooked mechanism behind race/ethnic disparities. This is the very first study on MDRs of educational attainment on youth social, emotional, and behavioral outcomes. 

## 6. Conclusions 

Compared to non-Hispanic Whites, Hispanic and Black adolescents maintain at high risk of social, emotional, and behavioral problems, despite high parental educational attainment. The benefits of parental education attainment on adolescents’ social, emotional, and behavioral problems are not equally seen by race and ethnic groups. Factors such as racism, segregation, stratification, and discrimination reduce Black and Hispanic families’ abilities to leverage their education and secure outcomes. Clinicians, policy makers, and researchers should all be aware that parental educational attainment consistently generates less outcomes for the privileged than the under-privileged groups. More research should be dedicated to exploring the contextual and societal causes of MDRs. Policy solutions should also address the societal and environmental barriers that are common in the daily lives of Hispanic and Black families and limit the benefits that educational attainment can generate for adolescents.

## Figures and Tables

**Table 1:** Measurements and Coding.

Variable	Role	Type	Measurement	Range	Coding	Reference
Age	Confounder	Continuous		8–11	-	-
Gender	Confounder	Categorical	Observed	0–1	Male = 1, Female = 0	-
Parental Marital Status	Confounder	Categorical	Parents Report	0–1	Married = 1 Other = 0	-
Parental Education	Independent Variable	Continuous	Parents Report	1–21	0 = Never attended/Kindergarten only; 1 = 1st grade; 2 = 2nd grade; 3 = 3rd grade; 4 = 4th grade 4; 5 = 5th grade; 6 = 6th grade 6; 7 = 7th grade 7; 8 = 8th grade; 9 = 9th grade; 10 = 10th grade 10; 11 = 11th grade; 12 = 12th grade; 13 = High school graduate; 14 = GED or equivalent Diploma; 15 = Some college; 16 = Associate degree: Occupational; 17 = Associate degree: Academic Program; 18 = Bachelor’s degree (ex. BA; 19 = Master’s degree (ex. MA; 20 = Professional School degree (ex. MD; 21 = Doctoral degree.	-
CBCL-anxious and depressed mood	Dependent Variable	Continuous	Parent Report	0–26	Items Responses: 0, 1, 2	CBCL [2]
CBCL-withdrawn and depressed affect	Dependent Variable	Continuous	Parent Report	0–14	Items Responses: 0, 1, 2	CBCL [2]
CBCL-social and interpersonal problems	Dependent Variable	Continuous	Parent Report	0–16	Items Responses: 0, 1, 2	CBCL [2]
CBCL-somatic complaints	Dependent Variable	Continuous	Parent Report	0–18	Items Responses: 0, 1, 2	CBCL [2]
CBCL-thought problems	Dependent Variable	Continuous	Parent Report	0–18	Items Responses: 0, 1, 2	CBCL [2]
CBCL-attention problems	Dependent Variable	Continuous	Parent Report	0–20	Items Responses: 0, 1, 2	CBCL [2]
CBCL-violent and aggressive behaviors	Dependent Variable	Continuous	Parent Report	0–38	Items Responses: 0, 1, 2	CBCL [2]
CBCL-rule-breaking behaviors	Dependent Variable	Continuous	Parent Report	0–38	Items Responses: 0, 1, 2	CBCL [2]

Child Behavior Checklist (CBCL).

**Table 2:** Descriptive data overall.

	All (*n* = 10,762)	
	*n*	%
**Race**		
Whites	8257	76.7
Blacks	2506	23.3
**Ethnicity**		
Non-Hispanic	9006	83.7
Hispanic	1757	16.3
**Gender**	5137	47.7
Male	5626	52.3
Female		
**Parental Marital Status**	3442	32.0
Other	7321	68.0
Married		
	**Mean**	**SD**
**Age (Year)**	9.48	0.51
**Parental Education**	16.74	2.60
**CBCL-** **A** **nxious and depressed mood (0–26)**	2.71	3.17
**CBCL-Withdrawn and depressed affect(0–14)**	1.05	1.72
**CBCL-Somatic complaints(0–16)**	1.48	1.94
**CBCL-Social and interpersonal problems(0–18)**	1.61	2.28
**CBCL-Thought problems(0–18)**	1.65	2.23
**CBCL-Rule-breaking behaviors (0–20)**	1.23	1.89
**CBCL-Attention problems (0–38)**	5.49	5.47
**CBCL-Violent and aggressive behaviors (0–38)**	3.45	4.55

CBCL = Child Behavior Checklist; SD = Standard Deviation.

**Table 3:** Bivariate correlations in the pooled sample.

	1	2	3	4	5	6	7	8	9	10	11	12	13
1 Race (African American)	1	−0.12 *	−0.02 *	−0.41 *	−0.28 *	−0.04 *	0.04 *	0.00	0.09 *	−0.01	0.14 *	0.04 *	0.06 *
2 Ethnicity (Hispanic)		1	−0.00	−0.08 *	−0.23 *	0.02	0.03 *	0.02 *	0.02 *	−0.02	−0.00	0.02	0.00
3 Gender (Male)			1	0.01	−0.00	0.02 *	0.05 *	−0.03 *	0.05 *	0.10 *	0.12 *	0.016 *	0.10 *
4 Parental Marital Status (Married)				1	0.36 *	−0.04 *	−0.10 *	−0.07 *	−0.14 *	−0.06 *	−0.18 *	−0.12 *	−0.13 *
5 Parental Education (Years)					1	−0.00	−0.10 *	−0.04 *	−0.13 *	−0.05 *	−0.17 *	−0.08 *	−0.10 *
6 Anxious and depressed mood,						1	0.58 *	0.47 *	0.62 *	0.60 *	0.41 *	0.57 *	0.58 *
7 Withdrawn and depressed affect							1	0.39 *	0.56 *	0.51 *	0.39 *	0.49 *	0.52 *
8 Somatic complaints								1	0.42 *	0.44 *	0.28 *	0.44 *	0.39 *
9 Social and interpersonal problems									1	0.61 *	0.55 *	0.69 *	0.67 *
10 Thought problems										1	0.51 *	0.73 *	0.63 *
11 Rule−breaking behaviors											1	0.65 *	0.73 *
12 Attention problems												1	0.76 *
13 Violent and aggressive behaviors.													1

* *p* < 0.01. Pearson Correlation Test.

**Table 4:** Summary of linear regressions overall.

	B	SE	95% CI		*p*	B	SE	95% CI		*p*
**Anxious and depressed mood**										
Race (Black)	−0.51	0.08	−0.67	−0.34	<0.001	−2.91	0.49	−3.87	−1.95	<0.001
Ethnicity (Hispanic)	0.04	0.09	−0.13	0.21	0.677	−1.55	0.47	−2.47	−0.62	0.001
Gender (Male)	0.12	0.06	0.00	0.24	0.049	0.11	0.06	0.00	0.23	0.060
Age	−0.07	0.06	−0.19	0.05	0.226	−0.08	0.06	−0.20	0.04	0.190
Parental Marital Status (Married)	−0.47	0.07	−0.61	−0.32	<0.001	−0.47	0.07	−0.62	−0.33	<0.001
Parental Educational Attainment	0.01	0.01	−0.02	0.03	0.643	−0.06	0.02	−0.10	−0.03	0.001
Parental Educational Attainment × Race	-	-	-	-	-	0.15	0.03	0.09	0.21	0.000
Parental Educational Attainment × Ethnicity	-	-	-	-	-	0.09	0.03	0.04	0.15	0.001
**Withdrawn and depressed affect**										
Race (Black)	−0.06	0.04	−0.15	0.03	0.176	−0.56	0.27	−1.08	−0.04	0.034
Ethnicity (Hispanic)	0.05	0.05	−0.05	0.14	0.328	−0.10	0.25	−0.60	0.40	0.696
Gender (Male)	0.19	0.03	0.12	0.25	<0.001	0.18	0.03	0.12	0.25	0.000
Age	0.09	0.03	0.03	0.16	0.005	0.09	0.03	0.03	0.15	0.005
Parental Marital Status (Married)	−0.31	0.04	−0.39	−0.23	<0.001	−0.31	0.04	−0.39	−0.23	<0.001
Parental Educational Attainment	−0.05	0.01	−0.06	−0.04	<0.001	−0.06	0.01	−0.08	−0.04	0.000
Parental Educational Attainment × Race	-	-	-	-	-	0.03	0.02	0.00	0.06	0.050
Parental Educational Attainment × Ethnicity	-	-	-	-	-	0.01	0.02	−0.02	0.04	0.606
**Somatic complaints**										
Race (Black)	−0.16	0.05	−0.26	−0.06	0.001	−2.00	0.30	−2.59	−1.42	<0.001
Ethnicity (Hispanic)	0.05	0.05	−0.06	0.15	0.383	−0.72	0.29	−1.28	−0.15	0.013
Gender (Male)	−0.13	0.04	−0.20	−0.06	0.001	−0.13	0.04	−0.21	−0.06	<0.001
Age	0.03	0.04	−0.04	0.11	0.345	0.03	0.04	−0.04	0.10	0.415
Parental Marital Status (Married)	−0.33	0.05	−0.42	−0.24	<0.001	−0.34	0.05	−0.43	−0.25	<0.001
Parental Educational Attainment	−0.02	0.01	−0.03	0.00	0.031	−0.06	0.01	−0.08	−0.04	<0.001
Parental Educational Attainment × Race	-	-	-	-	-	0.11	0.02	0.08	0.15	<0.001
Parental Educational Attainment × Ethnicity	-	-	-	-	-	0.04	0.02	0.01	0.08	0.013
**Social and interpersonal problems**										
Race (Black)	0.11	0.06	0.00	0.22	0.059	−1.52	0.35	−2.21	−0.84	<0.001
Ethnicity (Hispanic)	−0.01	0.06	−0.13	0.11	0.868	−1.27	0.34	−1.93	−0.62	<0.001
Gender (Male)	0.25	0.04	0.16	0.33	<0.001	0.24	0.04	0.16	0.33	<0.001
Age	−0.13	0.04	−0.22	−0.05	0.002	−0.14	0.04	−0.22	−0.05	0.002
Parental Marital Status (Married)	−0.50	0.05	−0.60	−0.40	<0.001	−0.50	0.05	−0.61	−0.40	<0.001
Parental Educational Attainment	−0.08	0.01	−0.09	−0.06	<0.001	−0.13	0.01	−0.15	−0.10	<0.001
Parental Educational Attainment × Race	-	-	-	-	-	0.10	0.02	0.06	0.14	<0.001
Parental Educational Attainment × Ethnicity	-	-	-	-	-	0.08	0.02	0.04	0.12	<0.001
**Thought problems**										
Race (Black)	−0.24	0.06	−0.35	−0.13	<0.001	−1.17	0.34	−1.84	−0.49	0.001
Ethnicity (Hispanic)	−0.23	0.06	−0.35	−0.12	<0.001	−2.13	0.33	−2.78	−1.49	<0.001
Gender (Male)	0.45	0.04	0.37	0.53	<0.001	0.45	0.04	0.36	0.53	<0.001
Age	−0.04	0.04	−0.12	0.04	0.354	−0.04	0.04	−0.12	0.04	0.338
Parental Marital Status (Married)	−0.29	0.05	−0.39	−0.19	<0.001	−0.28	0.05	−0.39	−0.18	<0.001
Parental Educational Attainment	−0.04	0.01	−0.06	−0.03	<0.001	−0.09	0.01	−0.12	−0.07	<0.001
Parental Educational Attainment × Race	-	-	-	-	-	0.05	0.02	0.01	0.09	0.011
Parental Educational Attainment × Ethnicity	-	-	-	-	-	0.12	0.02	0.08	0.16	<0.001
**Rule−breaking behaviors**										
Race (Black)	0.25	0.05	0.16	0.34	<0.001	−0.64	0.28	−1.20	−0.08	0.025
Ethnicity (Hispanic)	−0.15	0.05	−0.25	−0.05	0.002	−1.60	0.27	−2.13	−1.06	<0.001
Gender (Male)	0.44	0.04	0.37	0.51	<0.001	0.44	0.04	0.37	0.51	<0.001
Age	−0.07	0.03	−0.13	0.00	0.057	−0.07	0.03	−0.14	0.00	0.051
Parental Marital Status (Married)	−0.49	0.04	−0.57	−0.41	<0.001	−0.48	0.04	−0.57	−0.40	<0.001
Parental Educational Attainment	−0.08	0.01	−0.10	−0.07	<0.001	−0.13	0.01	−0.15	−0.10	<0.001
Parental Educational Attainment × Race	-	-	-	-	-	0.05	0.02	0.02	0.09	0.003
Parental Educational Attainment × Ethnicity	-	-	-	-	-	0.09	0.02	0.06	0.12	0.000
**Attention problems**										
Race (Black)	−0.14	0.14	−0.41	0.13	0.308	−4.24	0.83	−5.87	−2.62	<0.001
Ethnicity (Hispanic)	−0.05	0.15	−0.34	0.24	0.725	−4.84	0.80	−6.40	−3.28	<0.001
Gender (Male)	1.79	0.10	1.59	2.00	<0.001	1.78	0.10	1.58	1.98	<0.001
Age	−0.37	0.10	−0.57	−0.17	<0.001	−0.38	0.10	−0.58	−0.18	<0.001
Parental Marital Status (Married)	−1.28	0.13	−1.53	−1.03	<0.001	−1.27	0.13	−1.52	−1.03	<0.001
Parental Educational Attainment	−0.10	0.02	−0.14	−0.05	<0.001	−0.26	0.03	−0.32	−0.19	<0.001
Parental Educational Attainment × Race	-	-	-	-	-	0.25	0.05	0.15	0.35	<0.001
Parental Educational Attainment × Ethnicity	-	-	-	-	-	0.29	0.05	0.20	0.39	<0.001
**Violent and aggressive behaviors**										
Race (Black)	−0.08	0.12	−0.31	0.14	0.463	−2.15	0.69	−3.51	−0.79	0.002
Ethnicity (Hispanic)	−0.26	0.12	−0.50	−0.02	0.033	−2.99	0.67	−4.30	−1.68	<0.001
Gender (Male)	0.96	0.09	0.79	1.13	<0.001	0.95	0.09	0.78	1.12	<0.001
Age	−0.23	0.09	−0.39	−0.06	0.007	−0.23	0.09	−0.40	−0.07	0.006
Parental Marital Status (Married)	−1.06	0.11	−1.27	−0.85	<0.001	−1.05	0.11	−1.26	−0.85	<0.001
Parental Educational Attainment	−0.12	0.02	−0.16	−0.09	<0.001	−0.21	0.03	−0.26	−0.16	<0.001
Parental Educational Attainment × Race	-	-	-	-	-	0.12	0.04	0.04	0.21	0.004
Parental Educational Attainment × Ethnicity	-	-	-	-	-	0.17	0.04	0.09	0.25	<0.001

B = Unstandardized Regression Coefficient; SE = Standard Error; CI = Confidence Interval.
